# Supra-Optimal Temperature: An Efficient Approach for Overaccumulation of Starch in the Green Alga *Parachlorella kessleri*

**DOI:** 10.3390/cells10071806

**Published:** 2021-07-16

**Authors:** Vilém Zachleder, Veronika Kselíková, Ivan N. Ivanov, Vitali Bialevich, Milada Vítová, Shuhei Ota, Tsuyoshi Takeshita, Shigeyuki Kawano, Kateřina Bišová

**Affiliations:** 1 Laboratory of Cell Cycles of Algae, Centre Algatech, Institute of Microbiology of the Czech Academy of Sciences, 37981 Třeboň, Czech Republic; zachleder@alga.cz (V.Z.); kselikova@alga.cz (V.K.); ivanov@alga.cz (I.N.I.); bialevich@alga.cz (V.B.); vitova@alga.cz (M.V.); 2 Faculty of Science, University of South Bohemia, 37005 České Budějovice, Czech Republic; 3 Center for Environmental Biology and Ecosystem Studies, National Institute for Environmental Studies, Tsukuba 305 8506, Ibaraki, Japan; ota.shuhei@nies.go.jp; 4 The University of Tokyo Future Center Initiative, Wakashiba 178 4 4, Kashiwa 277 0871, Chiba, Japan; takeshita@algalbio.co.jp (T.T.); kawano@edu.k.u-tokyo.ac.jp (S.K.)

**Keywords:** microalgae, *Parachlorella kessleri*, starch, supra-optimal temperature, cell cycle, energy reserves, growth processes, reproduction events, deuterium, deuterated starch, deuterated lipid

## Abstract

Green algae are fast-growing microorganisms that are considered promising for the production of starch and neutral lipids, and the chlorococcal green alga *Parachlorella kessleri* is a favorable model, as it can produce both starch and neutral lipids. *P. kessleri* commonly divides into more than two daughter cells by a specific mechanism—multiple fission. Here, we used synchronized cultures of the alga to study the effects of supra-optimal temperature. Synchronized cultures were grown at optimal (30 °C) and supra-optimal (40 °C) temperatures and incident light intensities of 110 and 500 μmol photons m^−2^ s^−1^. The time course of cell reproduction (DNA replication, cellular division), growth (total RNA, protein, cell dry matter, cell size), and synthesis of energy reserves (net starch, neutral lipid) was studied. At 40 °C, cell reproduction was arrested, but growth and accumulation of energy reserves continued; this led to the production of giant cells enriched in protein, starch, and neutral lipids. Furthermore, we examined whether the increased temperature could alleviate the effects of deuterated water on *Parachlorella kessleri* growth and division; results show that supra-optimal temperature can be used in algal biotechnology for the production of protein, (deuterated) starch, and neutral lipids.

## 1. Introduction

Light and temperature are two crucial factors affecting algal growth and division, both in natural habitats and in biotechnological applications. For autotrophically grown organisms, there is a physiological range of light intensities and temperatures that support growth and division, and light intensities and temperatures below the threshold are not sufficient. On the other hand, light intensities and temperatures above this range are increasingly stressful, and will inhibit cell division and/or growth. The duration of light, along with its intensity and spectral composition will, in autotrophically grown algae, affect growth rates [1,2,3,4,5,6,7,8,9,10,11], but will not affect light-independent processes such as cell division, which uses internal energy stores for its support [1,5,6,7,8,9,11]. In contrast, temperature is much less specific, as it will affect all metabolic processes, including “dark” ones such as cell division; thus, its effects reach beyond setting growth rates. In principle, within the physiological range, a 10 °C increase in temperature will increase metabolic rate twofold [12]. Increasing temperature will thus speed up growth, as well as individual processes leading to cell division, and consequently shorten the duration of the cell cycle, as shown in different algae such as *Chlorella ellipsoidea* [11], *Chlamydomonas reinhardtii* [13], *Chlamydomonas eugametos* [14], and *Desmodesmus quadricauda* [15]. However, individual metabolic processes are differentially sensitive to various stresses [16], including temperature [17], which significantly affects how cells react to changes in temperature. In particular, cell division seems to be more sensitive to temperature increases than growth does [17,18]; this has peculiar consequences; there are threshold temperatures that will only slightly or not at all affect cell growth, but will completely block cell division. The effects of such supra-optimal temperatures were described in the green algae *Chlamydomonas reinhardtii* [17] and *Chlorella vulgaris* [18]. 

The chlorococcal alga *Parachlorella kessleri* (formerly *Chlorella kessleri* [19]) is a biotechnologically promising algal species [20,21,22,23,24,25,26,27,28,29] that divides by multiple fission. Its cell cycle consists of multiple rounds of DNA replication that, after completion of the last one, are followed by successive nuclear divisions. Each of the nuclear divisions is immediately followed by cell division. Morphologically, the cells become sequentially polyploid but not polynuclear [30]. Within a single cell cycle, the mother cell can give rise to 2, 4, 8, or 16 cells; this is consistent with the established scheme of multiple fission, where a single mother cell can divide into 2*^n^* daughter cells—where *n* is the number of rounds of started reproductive sequences (DNA replication, nuclear division, cell division) [31]. Increasing light intensity will increase both growth rate and the number of reproductive sequences started, and thus, the number of daughter cells being formed from a single cell [30]. 

The primary energy and carbon store of *P. kessleri* is starch, which is produced under optimal growth conditions in complete nutrient medium and at physiological values of light intensity and temperature [23,32]. Starch is exclusively located in the chloroplasts in the form of starch grains of different numbers and sizes. In the autotrophically grown algal cell, starch reserves serve mostly as a buffer to supply a stable carbon and energy source. This is indispensable, as in nature the energy supply from photosynthesis varies with sunlight intensity during the day, and is absent during nights. The buffering role of starch is crucial for cell reproduction. The processes of DNA replication, along with nuclear and cellular divisions, are of importance in the life of a cell and so they depend on a constant and reliable supply of energy and carbon. Indeed, the majority of starch produced by cells is degraded exclusively for cell reproduction [33], and this is true even for cells growing in continuous light [34]. This was evidenced in synchronized cultures of different algal species, where net starch increased to some maximal value during the cell cycle, and subsequently was nearly completely degraded during nuclear and cellular division at the end of the cell cycle [33,35,36]. The cellular starch content at any given time is the net result of starch synthesis and utilization; thus, decreasing starch content can be caused by lower starch synthesis, by increased consumption, or by a combination of both factors. It seems that a decrease in starch content at the time of cell division is caused by a combination of increased starch spending for cell reproduction and a minimum photosynthetic rate specific for this period [35]. Furthermore, a blockage of cell division processes as the primary starch consumers will lead to starch (over)accumulation. This has been established for different stress conditions, such as the application of inhibitors [36], nutrient starvation [24,25,32,36], high light intensity [32], the presence of a high concentration of CO_2_ [37], or supra-optimal temperatures [18], and it should be explored biotechnologically [36,38]. If stress conditions are prolonged, starch reserves will gradually start to be replaced by neutral lipids—the secondary energy store of *P. kessleri* [23,24,32,39]. Although neutral lipids under optimal growth conditions are maintained at a relatively low level, at 1–10% of dry matter (DM) [23,39,40], they can also (over)accumulate under stress conditions similar to those inducing starch accumulation, such as nitrogen, sulfur, or phosphorus depletion [24,26,32,39,41], dilution of all the nutrients in the medium [25,39] or salt stress [42]. The ability to (over)produce both starch and neutral lipids makes *P. kessleri* a useful model for applications in biotechnology. A peculiar source of stress for *P. kessleri* is cultivation in deuterium, which leads predominantly to the accumulation of starch [30], but also that of neutral lipids [43]. Deuterium—the stable isotope of hydrogen—is known to have the highest kinetic effect among the stable isotopes of biogenic elements [43,44,45]. Deuterium in deuterated water is known to increase cell stress via several mechanisms [43,44], including greater bond dissociation and activation energies [46]. Its presence affects the entire metabolism, and its specific effects range from disrupting signaling [47] and energy production in mitochondria and chloroplasts [48,49] to disrupting cell division [50,51]. Given the very low concentrations of deuterium (or deuterated water) in nature, its application in high concentrations is artificial, and is also quite expensive. However, deuterated molecules are used extensively as analytical standards and for metabolic labeling [43,52,53], and deuterated compounds are exploited in pharmacology [44,54,55,56,57]. Therefore, production of algal-derived deuterated compounds is justified for the production of fine (bio)chemicals with very high added value. Indeed, algal-derived deuterated biomolecules such as sugars, proteins, carotenoids, lipids, and starch can be used commercially [43,52,53,58,59,60].

Here, we supplement the extensive knowledge that has accumulated on starch and neutral lipid production under different conditions in *P. kessleri* by providing baseline information on the effects of supra-optimal temperature on cell reproduction (DNA replication, cell division) and growth (RNA, protein, dry matter, cell volume). Particular focus has been placed on the production of starch and lipids as the primary and secondary energy stores. Furthermore, we analyze the effects of a high concentration of deuterium on cell growth and reproduction, as well as the production of deuterated starch and lipids. The results of this study can be exploited as a starting point for further optimization in larger scale biotechnological production, but will also widen the *P. kessleri* portfolio as a model for basic research. 

## 2. Materials and Methods

### 2.1. Organism and Culture

The green unicellular microalga *Parachlorella kessleri* (Trebouxiophyceae, Chlorophyta) (strain CCALA 255) was obtained from the Culture Collection of Autotrophic Organisms at the Institute of Botany, Czech Academy of Sciences in Třeboň, Czech Republic (CCALA; https://ccala.butbn.cas.cz/, accessed on 7 June 2021).

For routine sub-culturing, the cultures were streaked every three weeks onto nutrient medium (see below) solidified by agar (1.5%), and grown on a light shelf at an incident light intensity of 100 μmol photons m ^−^^2^ s ^−^^1^ of photosynthetically active radiation.

### 2.2. Mineral Nutrient Medium

The mineral medium was based on the mean content of P, N, K, Mg, and S in algal biomass [61], and had the following initial composition (in mg/L): 1100 KNO_3_, 237 KH_2_PO_4_, 204 MgSO_4_.7H_2_O, 40 C_10_H_12_O_8_N_2_NaFe, 88 CaCl_2_, 0.83 H_3_BO_3_, 0.95 CuSO_4_.5H_2_O, 3.3 MnCl_2_.4H_2_O, 0.17 (NH_4_)_6_Mo_7_O_24_.4H_2_O, 2.7 ZnSO_4_.7H_2_O, 0.6 CoSO_4_.7H_2_O, and 0.014 NH_4_VO_3_ in distilled water [36]. For the preparation of the medium, 100x concentrated stock solutions of macroelements and microelements were used. All components were diluted in distilled water and autoclaved for 30 min at 121 °C. The pH was adjusted to 7 with 1 M NaOH.

### 2.3. Culture Conditions

Two types of cultivation units were used for experiments: (1) glass cylinders (inner diameter 36 mm, height 500 mm, volume of suspension 300 mL), and (2) flat and rectangular glass vessels (inner dimensions 400 × 300 × 20 mm, volume of suspension 2500 mL). The first type of the units was used for routine growth, synchronization, and small-scale experiments with deuterated water; the second type of the units was used for large-scale experiments. Culture units were placed in a thermostatic water bath, where the temperature was set and automatically maintained at the values stated in the Results section for each experiment. The vessels were illuminated from one side with a panel of dimmable fluorescent lamps (DULUX L55W/950 Daylight, OSRAM, Munich, Germany). For the experiments, the incident light intensity was set either to 110 or 500 μmol photons m ^−^^2^ s ^−^^1^ of photosynthetically active radiation. The cultures were vigorously mixed with air bubbles containing 2% carbon dioxide (*v*/*v*) dispersed through a 200-μL micropipette tip fixed in a glass tube placed at the bottom of the cylinder. The aeration in the flat vessels was performed via bubble stream from a perforated stainless steel tube located at the bottom of the vessel. The flow rate of the aeration mix was 60 L/h.

### 2.4. Synchronization of Cultures

Cultures were synchronized under optimal conditions: incident light intensity 500 μmol photons m ^−2^ s ^−1^, temperature 30 °C, 2% CO_2_ (*v*/*v*) in aeration mixture. The cultures were initiated by inoculating algal cells directly from the culture plates into the liquid medium. Such cultures were synchronized by alternation of 18 h light and 7 h of dark, as established earlier [30]. During the synchronization procedure, the cell density was kept below 1 × 10^6^ cells mL^−1^ by dilution at the end of the dark period, in order to prevent cell shading. This regime was maintained for several days (about 3 cycles) until the required culture synchrony was reached. Once the cultures were synchronized, they were grown for approximately one more cycle at the same cell density until the volume of the culture required for the start of the experiment was reached. The synchronized daughter cells were again diluted to the initial cell density of approximately 1 × 10^6^ cells mL^−1^, and used as inocula for experimental cultures.

### 2.5. Management of Deuterated Cultures

The synchronized inocula for the deuterated water experiments were centrifuged at 3000× *g* for 5 min and resuspended in a medium containing the desired concentration of deuterated water. The cultures were cultivated in a semi-batch mode, controlled by the optical density at 750 nm (OD_750_). Upon reaching an OD_750_ of 0.4, the cultures were diluted with fresh medium with corresponding D_2_O content to the initial value of OD_750_. This way, growth unlimited by nutrients and light was achieved.

### 2.6. Measurement of Light Intensity

The dimmable fluorescent tubes were used for the adjustment of the incident irradiance. The light intensity was measured using a quantum/radiometer/photometer (LI-COR, Inc., Lincoln, NE, USA). Incident light intensity (I_i_) was measured at the surface of the culture vessel, while the transmitted light intensity was measured at the rear side of the culture vessel (I_t_). The different values of incident (I_i_) and transmitted (I_t_) light intensities at different optical densities given by cell size and concentrations were used to calculate the mean light intensity (I_m_)—i.e., light energy absorbed by a layer of cell suspension—according to the Lambert–Beer formula: I_m_ = (I_i_ − I_t_)/ln(I_i_/I_t_).

### 2.7. Assessment of Cell Division Curves

The proportion of mother cells and daughter cells was determined by light microscopy in cells fixed in Lugol’s solution (1 g I, 5 g KI, 100 mL H_2_O) at a final concentration of 10 μL of Lugol’s solution per 1 mL of cell suspension.

### 2.8. Dry Matter Determination

Biomass was separated from the medium by centrifugation of 4 mL of the cell suspension in pre-weighed microtubes at 3000× *g* for 5 min; the sediment was dried at 105 °C for 12 h and weighed on an analytical balance (TE214S-0CE, Sartorius, Goettingen, Germany) [36].

### 2.9. Cell Volume and Number

Cell volume and number were measured using a Beckman Coulter Multisizer 4 (Beckman Coulter Life Sciences, Indianapolis, IN, USA) by diluting 50 μL of fixed (0.2% glutaraldehyde) cell suspension into 10 mL of 0.9% NaCl (*w*/*v*) electrolyte solution.

### 2.10. Quantum Yield Measurement

Aliquots of 2 mL were withdrawn from the culture and placed into 10 × 10-mm plastic cuvettes for 30 min in the dark. Quantum yield was measured using an AquaPen-C 100 (Photon Systems Instruments, Drasov, Czech Republic).

### 2.11. Neutral Lipids and Starch Staining

For neutral lipids and starch staining, 1 mL of culture was sampled and stored for up to several hours at 4 °C until analysis. Staining was conducted on microscopy slides prior to observation. For starch staining, 20 μL of culture was combined with 0.5 μL of Lugol’s solution (1 g I, 5 g KI, 100 mL H_2_O) and observed immediately via light microscope (model BX51, Olympus, Tokyo, Japan). Neutral lipids were stained with the addition of 0.5 μL of freshly prepared Nile red dye (0.5 mg/mL in DMSO, catalog no. 72485, Sigma-Aldrich, Prague, Czech Republic) to 20 μL of culture, directly on the microscopic slide. Such slides were incubated at room temperature for 20 min in the dark. Stained cells were observed using a microscope (model BX51, Olympus, Tokyo, Japan) equipped with a mercury lamp and filter combination U-MNU2 (360–370 nm excitation and >515 nm barrier filter). Microphotographs were taken with a DP72 camera (Olympus, Tokyo, Japan).

### 2.12. Neutral Lipids Quantification

Neutral lipids content was measured spectrophotometrically in a microplate format following the modified procedure of Takeshita, et al. [62]. Aliquots (100 μL) of the cultures were combined with 5 μL of freshly prepared Nile red dye (0.5 mg/mL in DMSO, catalog no. 72485, Sigma-Aldrich, Prague, Czech Republic) in a 96-well plate. The same amount of Nile red dye was added to a sample blank consisting of 100 μL of H_2_O. The plate was incubated at room temperature in the dark for 15 min. Fluorescence was measured using an Infinite 200 PRO microplate reader (Tecan, Männedorf, Switzerland) equipped with a 485-nm excitation filter and a 595-nm emission filter. The fluorescence intensity of the samples was normalized using the fluorescence intensity of unstained samples and a blank. Standard curves produced from a commercial lipid standard—triolein (catalog no. Y0001113, Sigma-Aldrich, St. Luis, MO, USA)—were used to quantify neutral lipids.

### 2.13. Estimation of Bulk RNA, DNA, and Proteins

#### 2.13.1. Total Nucleic Acids Extraction

The procedure of Wanka [63], as modified by Lukavský, et al. [64], was used for the acidic extraction of total nucleic acids. After the removal of small-molecular-weight contaminants, the total nucleic acids were hydrolyzed with 0.5 M perchloric acid, and the absorbance of total nucleic acids in the supernatant was read at 260 nm (A_260_). Total nucleic acid concentration was calculated based on calibration with a DNA standard of known concentration treated using the same procedure, and normalized to the number of cells in the sample. 

#### 2.13.2. DNA and RNA Determination

The light-activated reaction of diphenylamine with hydrolyzed DNA, as described by Decallonne and Weyns [65], was used with the modifications of Zachleder [66]. The concentrations of DNA within the samples were set by comparison to the A_600_ and A_700_ nm values of the sample with known DNA concentration treated using the same procedure. The values were normalized to the number of cells extracted; the RNA content was calculated as the difference between the total nucleic acid and DNA contents.

#### 2.13.3. Protein Determination

The sediment remaining after nucleic acid extraction was used for protein determination; it was hydrolyzed with 1M NaOH for 1 h at 70 °C. The protein concentration in the supernatant after centrifugation of the hydrolysate (15 min, 5300× *g*, room temperature) was estimated by BCA assay (cat. no. 23225, Thermo Fisher Scientific, Waltham, MA, USA) according to the manufacturer’s specifications. The same procedure was carried out with a calibration curve set by different concentrations of bovine serum albumin.

### 2.14. Starch Analyses

Net starch content was determined as described by Zachleder, et al. [30]. In short, the cells were disintegrated by vortexing with zirconium beads, and the pigments were removed via the addition of 80% (*v*/*v*) ethanol to the pellet, followed by incubation for 15 min at 68 °C. The procedure was repeated until the pellets were completely discolored. After that, 1 mL of α-amylase from porcine pancreas (Sigma-Aldrich, St. Luis, MO, USA), (0.5 mgl^−1^ (*w*/*v*) in 0.1 M sodium phosphate buffer (pH 6.9)) was added to the samples, and they were incubated for 1 h at 37 °C (FTC 90i, VELP Scientifica, Usmate Velate MB, Italy). Starch content was determined in the supernatant using DNSA color reaction for the quantification of reducing sugars [67]. The concentration of starch was estimated through a calibration curve of potato starch (Lach-Ner, Neratovice, Czech Republic) digested with α-amylase. 

### 2.15. Statistical Analysis

Experiments were performed in at least three biological replicates, and the mean values were used to construct the graphs in SigmaPlot version 11 (Jandel Scientific Software, Erkrath, Germany). Regression curves of various orders were applied to fit the data. If not stated otherwise, all results are presented as means and standard deviation (*n* = 3). MS Excel 2016 was used to compute one- and two-way ANOVA. A *p*-value < 0.05 was considered to be significant.

## 3. Results

### 3.1. Growth in Normal Water

**Figure 1:**
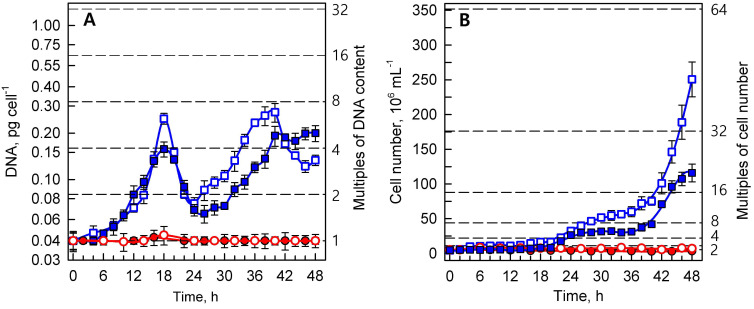
The course of reproductive events in the synchronized cultures of *Parachlorella kessleri* grown at temperatures of 30 °C and 40 °C (blue and red symbols and lines, respectively), and at incident light intensities of 500 and 110 μmol photons m^−2^ s^−1^ (empty and full symbols, respectively). (**A**) DNA (pg cell^−1^), and (**B**) cell number (10^6^ mL^−1^). Horizontal dashed lines indicate the number of doublings of the initial values at the beginning of the cell cycle (0 h). Multiples of these values are given on the right ordinate. Data are presented as means ± SE. The differences between 30 °C and 40 °C are statistically significant at *p* < 0.001 (two-way ANOVA) in both A and B.

**Figure 2:**
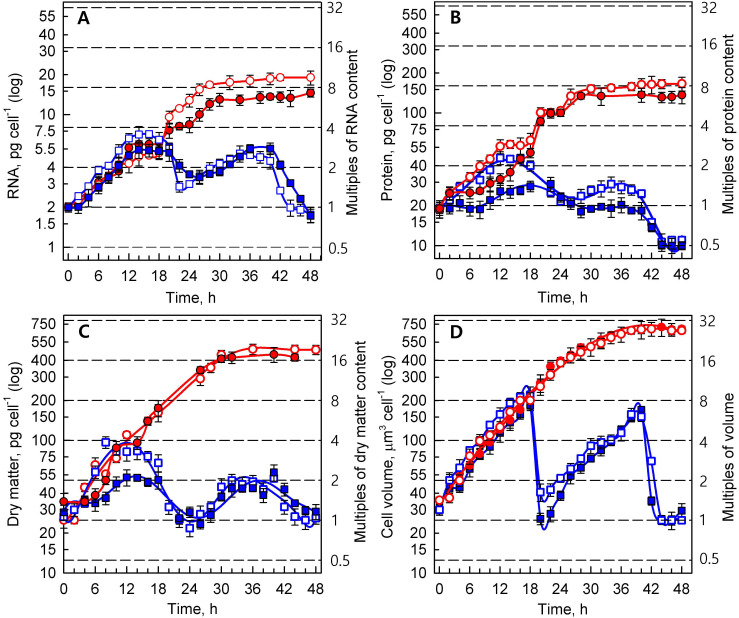
Time course of growth events in synchronized cultures of *Parachlorella kessleri* grown at 30 °C and 40 °C (blue and red symbols and lines, respectively), and at incident light intensities of 500 and 110 μmol photons m^−2^ s^−1^ (empty and full symbols, respectively). (**A**) RNA (pg cell^−1^), (**B**) protein (pg cell^−1^), (**C**) dry matter (pg cell^−1^), and (**D**) cell volume (μm^3^ cell^−1^). Horizontal dashed lines indicate the number of doublings of the initial values at the beginning of the cell cycle (0 h). Multiples of these values are given at right ordinate. Data are presented as means ± SE. The differences between 30 °C and 40 °C are statistically significant at *p* < 0.001 (two-way ANOVA) in both A and B.

**Figure 3:**
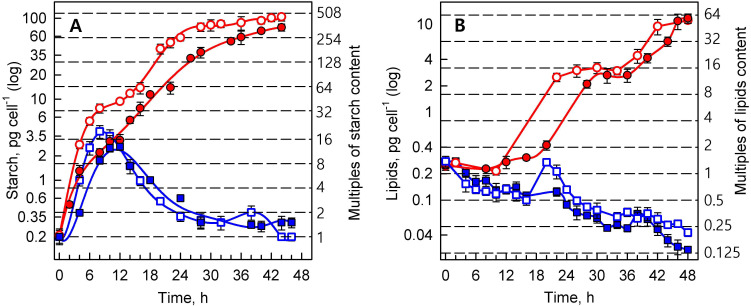
The course of changes in energy reserves in synchronized cultures of *Parachlorella kessleri* at temperatures of 30 °C and 40 °C (blue and red symbols and lines, respectively), and at incident light intensities of 500 and 110 μmol photons m^−2^ s^−1^(empty and full symbols, respectively). (**A**) Starch (pg cell^−1^), and (**B**) lipids (pg cell^−1^). Horizontal dashed lines indicate the number of doublings of the initial values at the beginning of the cell cycle (0 h). Multiples of these values are given on the right ordinate. Data are presented as means ± SE. The differences between 30 °C and 40 °C are statistically significant at *p* < 0.001 (two-way ANOVA) in both A and B. The differences between different light intensities in A are statistically significant at *p* < 0.05 (two-way ANOVA).

Synchronized cultures of *Parachlorella kessleri* were grown at optimal (30 °C) and supra-optimal (40 °C) temperatures, at low (110 μmol photons m^−2^ s^−1)^ and high (500 μmol photons m^−2^ s^−1^) incident light intensities. The reproductive events (DNA replication, cellular division) (Figure 1), growth processes (RNA and protein synthesis, dry matter, and cell volume) (Figure 2), and accumulation of energy reserves (starch and lipids) (Figure 3) were followed within the time interval corresponding to two consequent cell cycles (48 h) in the control culture. 

#### 3.1.1. Reproductive Events

Daughter cells that were released during the dark period were uninucleate, containing about 0.04 pg of DNA per nucleus. At the optimal temperature, the first replication round started after about 8 h and was completed at the 12^th^ h of growth in light at both light intensities. The second replication round was terminated by the 18^th^ h, when cell division started. The extent of multiplication differed between the two light intensities, as the DNA content increased about fourfold at low light intensity, and almost eightfold at high light intensity. The content of DNA per cell became correspondingly reduced during cell division between the 20^th^ and 24^th^ hours, when the cell numbers increased about eightfold and fourfold at high and low light intensities, respectively (Figure 1A). In the second cell cycle, the completion of DNA replication again shortly preceded the division of cells, attaining a maximum DNA content per cell just before the release of the daughter cells (Figure 1A,B). At high light intensity, DNA replication occurred earlier and to a greater extent than at the low light intensity. Similarly to the first cell cycle, nearly three replication rounds of DNA occurred at high light intensity, followed by division into mostly eight daughters, while at lower light intensity only two replication rounds and division into four daughter cells occurred during the cell cycles (compare Figure 1A,B). 

The reproductive events were completely inhibited in the cultures grown at the supra-optimal temperature (40 °C), which was demonstrated by constant values of DNA content (Figure 1A), and number of cells (Figure 1B) corresponding to the initial values at the beginning of the first cell cycle. 

#### 3.1.2. Growth Processes

Growth was characterized as changes in the cellular content (pg cell^−1^) of total RNA (Figure 2A), proteins (Figure 2B), dry matter (Figure 2C), and cell volume (μm^3^ cell^−1^) (Figure 2D). Total RNA content (Figure 2A) in the first cell cycle increased approximately threefold at the low light intensity and almost fourfold at high light intensity within 18 h of growth in cells grown at 30 °C, reaching a maximum just before cellular division, and then decreasing with time as the cells divided to a value corresponding to the RNA content of the daughter cells (Figure 2A). A similar behavior was also noted in the second cell cycle, with the maximum being reached after 36 h, i.e., 18 h after cell division started. However, the rate of RNA content increase was slower in the second cell cycle than in the first one, probably due to a decrease in the mean light intensity (Table 1) due to an increase in cell concentration by the end of the first cell cycle (Figure 2A). 

Similar kinetics were also observed in the time courses of protein content (Figure 2B), dry matter (Figure 2C), and cell volume (Figure 2D) at 30 °C. The growth characteristics (RNA, protein, and dry matter) were slowed, and attained a lower maximum content at the lower light intensity of 110 μmol photons m^−2^ s^−1^ than at the higher intensity of 500 μmol photons m^−2^ s^−1^. Within the second cell cycle, the differences between these variants were less apparent, and growth rate was suppressed by an increase in cell concentration, and a consequent decrease in mean light intensity (Table 1). Cell volume multiplied about eightfold (Figure 2D) in proportion to the number of daughter cells liberated at the end of both cell cycles at both light intensities (Figure 1B).

Completely different kinetics of growth processes occurred in cultures grown at 40 °C. As described above, the reproductive processes (events) at this temperature were inhibited (Figure 1), but the growth processes continued undisturbed for the duration of two cell cycles of the control cultures grown at 30 °C (Figure 2). The cells reached a giant size corresponding to about five doublings (32-fold increase) in cell volume within 36 h (Figure 2D). Within the same time period, protein content attained three doublings (8-fold increase; Figure 2B), dry matter attained four doublings (16-fold increase, Figure 2C), and RNA content increased 8-fold (Figure 2A). The proportions of different components of the cells differed compared to control cells.

#### 3.1.3. Energy Reserves

The biochemical analyses of starch content solely detect net starch, i.e., the total amount of starch produced in light minus starch consumed in light. Cells of the control culture grown at high light intensity increased their starch content to about 16 times the daughter cells’ content within 12 h of growth in the first cell cycle (Figure 3A). At the end of the cell cycle, the starch was extensively degraded, until it reached the values present in the daughter cells. At lower light intensity (110 μmol photons m^−2^ s^−1^), accumulation of starch to its maximal value during the growth phase was slower, and it attained a lower maximum than in the culture grown at high light intensity (500 μmol photons m^−2^ s^−1^) (Figure 3A). In the second cell cycle, the synthesis of starch decreased due to an increase in cell concentration caused by mother cell divisions (Figure 1B). This slowed the accumulation of starch so that it only covered the requirements of metabolism, but did not accumulate (Figure 3A). 

Due to inhibited reproductive processes at 40 °C, the net starch content did not decrease at the time of cell division in controls. Consequently, starch overaccumulated to between 508- and 254-fold compared to the initial content in the daughter cells, and to about 32-fold the maximum values found in the control cells (Figure 3A) The rate of accumulation at the lower light intensity was slightly slower, and the final content of overaccumulated starch was lower (77 vs. 104 pg cell^−1^) (Figure 3A). 

The secondary energy reserve, neutral lipids, did not accumulate at the optimal temperature of 30 °C. The lipid content remained fairly constant, and decreased during the second cell cycle (Figure 3B). Contrary to growth at 30 °C, lipid reserves at 40 °C increased from the middle of the first cell cycle until the end of the second cell cycle (Figure 3B). Lipid accumulation was delayed at low light intensity, but the final values—about a 64-fold increase compared to the initial values—were comparable between both light intensities (Figure 3B). 

### 3.2. Growth in Deuterated Water

**Figure 4:**
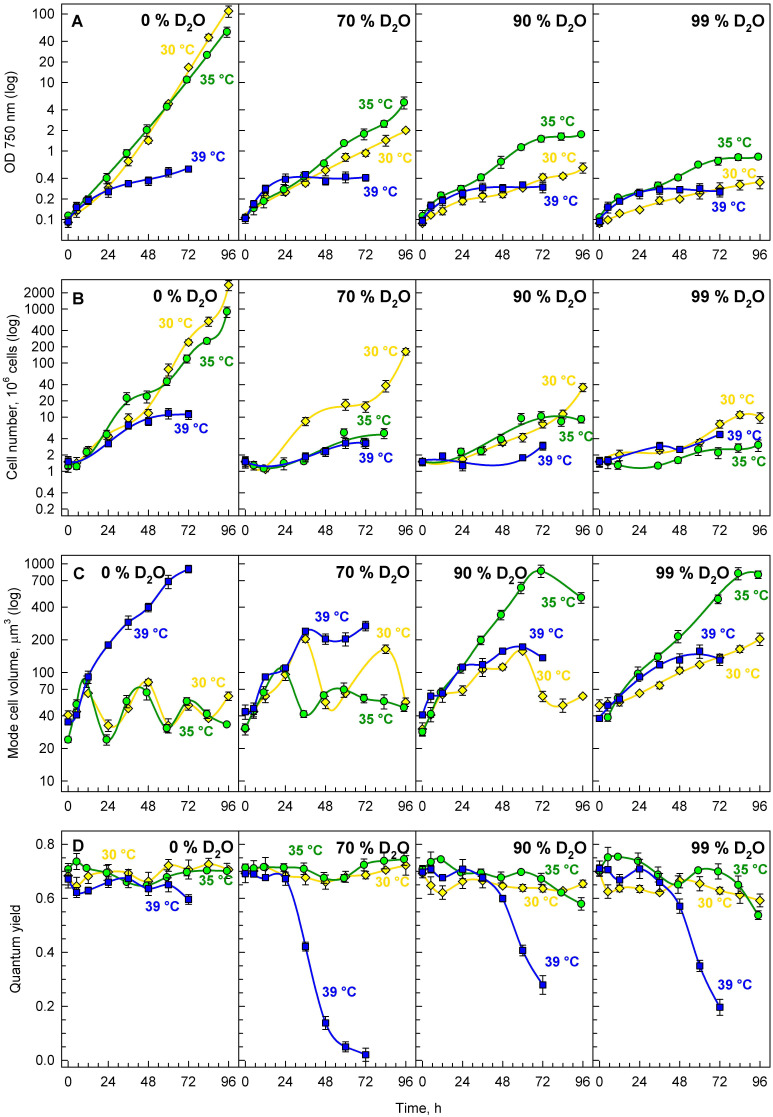
Time course of cell division and growth in synchronized cultures of *Parachlorella kessleri* grown at an incident light intensity of 110 μmol photons m^−2^ s^−1^ and temperatures of 30 °C (yellow diamonds), 35 °C (green circles), and 39 °C (blue squares), with different concentrations of deuterated water in the medium (0, 70, 90, and 99%). (**A**) Optical density at 750 nm, (**B**) cell number (10^6^ mL^−1^), (**C**) modal cell volume, and (**D**) quantum yield. Note the logarithmic scale on the Y-axis for (**A**–**C**). Note that the cultures were maintained in semi-batch mode. However, both panels (**A**) and (**B**) show recalculated values multiplied by individual dilution factors. Thus, they represent a projection of the growth in semi-batch cultivation mode to the continuous growth. This way, the plots remain easily readable.

Stress caused by growth at the supra-optimal temperature affected the cell composition, and significantly increased the content of both starch and lipids. Since increased temperature increases kinetic energy—leading to a seeming decrease in both dissociation and activation energies, which are both affected by the presence of deuterium—we tested whether the combination of increased temperature and the presence of deuterium would affect cell growth and accumulation of energy reserves. Synchronized cultures of *P. kessleri* were grown at the optimal temperature of 30 °C and two higher temperatures: 35 °C, and 39 °C. In contrast to the experiments described above, for the experiments in deuterated water, the temperature of 39 °C was used as the supra-optimal one; this was due to the fact that combination of the higher temperature (40 °C) and deuterated water strongly affected cell vitality and viability, but the effect of 39 °C on deuterated cultures (and control cultures) was similar to the effect of 40 °C. As high light intensity, in combination with the presence of deuterium, increases cellular stress [30], only a low light intensity of 110 μmol photons m^−2^ s^−1^ was used for these experiments. Due to the inhibitively high price of deuterated water, only cellular division was followed, and growth was assessed by changes in optical density at 750 nm (OD_750_) and cell volumes (Figure 4). The stress caused to the photosynthetic apparatus was estimated as quantum yield. 

#### 3.2.1. Growth and Division

Synchronous cultures of *P. kessleri* grown in deuterated water showed a concentration-dependent decrease in their growth rate (Figure 4A). Apart from decreased growth rates, changes in cell numbers (Figure 4B) indicated decreased division rates in deuterated cultures. This was further supported by observed changes in mode cell volume (Figure 4C), which reflected cell cycle progression in synchronous cultures. At 30 °C, the culture in 0% D_2_O (Figure 4C, first panel, yellow line) completed three full cell cycles, as illustrated by three sequences of cell volume increase and decrease, while there were only two cell cycles completed in 70% D_2_O (Figure 4C, second panel, yellow line), one full cell cycle in 90% D_2_O (Figure 4C, third panel, yellow line), and only growth—not followed by cell division—which was characteristic for the majority of cells in 99% D_2_O (Figure 4C, fourth panel, yellow line). 

Interestingly, the effects of temperature varied between different deuterated cultures. In the control culture, temperatures of 30 and 35 °C showed similar performance, both in terms of growth (Figure 4A, first panel) and cell cycle progression (Figure 4B,C, first panels), while at a temperature of 39 °C cell division was clearly inhibited, as documented by the occurrence of abnormally enlarged cells at 39 °C (Figure 4C, first panel, blue line), accompanied by significantly lower cell numbers (Figure 4B, first panel, blue line). This phenotype recapitulates what is described in more detail above. In deuterated cultures, the best performance, in terms of growth measured as OD_750_, was obtained at 35 °C (Figure 4A, green lines). However, such a performance was mainly caused by an increase in cell volume under such conditions—especially in 90% and 99% D_2_O (Figure 4C, green lines). At the same time, division was more adversely affected by cultivation at 35 °C (Figure 4B, green lines), as fewer cells were produced during cultivation. The adverse effect of cultivation at 39 °C was especially pronounced in quantum yields (Figure 4D, blue lines), which points to severe impairment of photosynthetic efficiency in such cultures.

In order to quantify and compare the effects of different deuterium concentrations (and their combination with tested temperatures), mass doubling time and cell number doubling time were calculated (Table 2). Mass doubling time increased with increasing D_2_O content in the growth medium, thus proving the adverse effect of D_2_O on growth. At the same time, cell number doubling time also increased with increasing D_2_O content in the medium, thus proving the adverse effect of deuterium on division. This trend applied to all temperatures tested; however, at 39 °C, it was the weakest, as such a temperature already poses severe stress—even for the control, undeuterated culture. Moreover, the effect of increasing D_2_O concentration on growth seemed to be more pronounced than the effect on cell division at 30 °C, but not at 35 and 39 °C (Table 2).

#### 3.2.2. Energy Reserves

**Figure 5:**
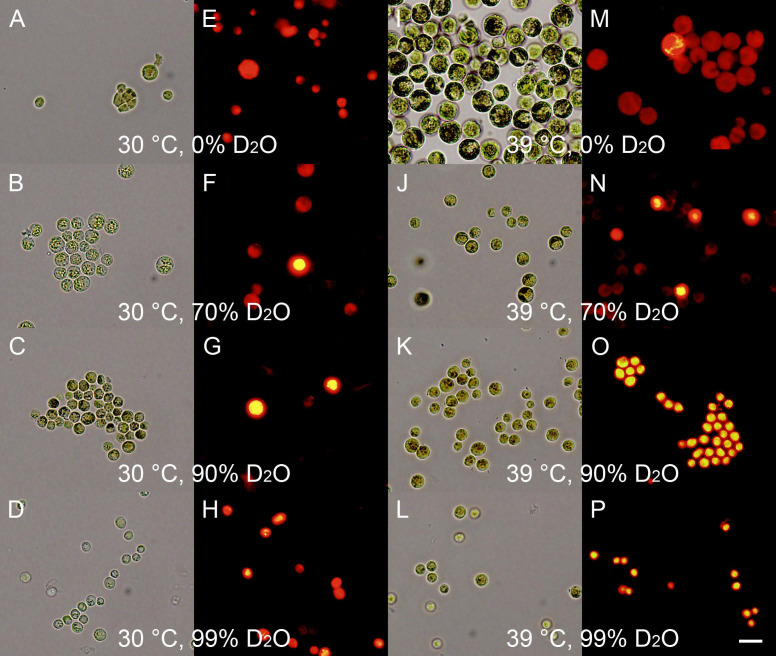
Light and fluorescence photomicrographs of synchronized cultures of *Parachlorella kessleri* grown for 24 h at an incident light intensity of 110 μmol photons m ^−2^ s ^−1^, temperatures of 30 °C and 39 °C, and different concentrations of deuterated water in the media (0, 70, 90, and 99%). (**A**,**E**,**I**,**M**) control cultures grown in 0% D_2_O, (**B**,**F**,**J**,**N**) cultures grown in 70% D_2_O, (**C**,**G**,**K**,**O**) cultures grown in 90% D_2_O, and (**D**,**H**,**L**,**P**) cultures grown in 99% D_2_O. In (**A**–**D**) and (**I**–**L**) the cells were stained with Lugol’s solution to visualize starch; for (**E**–**H**) and (**M**–**P**) the cells were stained with Nile red to visualize neutral lipids. Scale is 10 μm.

The combined effects of different deuterium concentrations with optimal and supra-optimal temperatures affected both starch and neutral lipid accumulation. After 24 h of cultivation, the starch content was increased in all variants except for the control culture grown at 30 °C (Figure 5, compare A with B–D and I–L). The degree of starch accumulation at 30 °C generally increased with increasing D_2_O concentration—except for 99% D_2_O, which contained the lowest amount of starch among cultures grown at 30 °C (Figure 5, compare panels A–D); at 39 °C, the net starch content was comparable in all variants (Figure 5I–L). The neutral lipids content was negligible in the control cultures grown in 0% D_2_O (Figure 5E), but it started to accumulate in some of the cells grown at 39 °C (Figure 5M), thus repeating the behavior described above (Figure 3). The deuterium-treated cultures had a higher content of neutral lipids, and their content increased with increases in both temperature and deuterium concentration from 70% D_2_O to 90% D_2_O (Figure 5). The cells grown at the highest deuterium concentration (99% D_2_O) contained slightly less neutral lipids than those grown in 90% D_2_O, but both of the cultures contained neutral lipids in almost all cells at 39 °C, which was in contrast to 70% D_2_O, where only about half of the cells contained detectable neutral lipids (Figure 5, compare O and P with N). 

## 4. Discussion

*Parachlorella kessleri* is an alga with emerging biotechnological potential, particularly because it can produce both starch as a primary energy store and neutral lipids as a secondary one [23,32]. Here, we used synchronized cultures of *P. kessleri* to analyze the effects of supra-optimal temperature on the production of starch and neutral lipids, both in the presence of normal (hydrogenated) water and of deuterated (heavy) water. We chose to use synchronized cultures where all of the cells within the cultures were in the same phase of the cell cycle. This allowed us to identify any subtle differences in the behavior of the supra-optimal-temperature-treated cultures and, thus, better characterize the effects. 

The application of different light intensities to the control culture at 30 °C led to different degrees of multiplication of DNA, and to the production of different numbers of daughter cells: four daughter cells at low light intensity, and eight daughter cells at high light intensity (Figure 1). Interestingly, the number of daughter cells produced was not significantly affected by the decreasing light intensities in the second cycle. At the low light intensity, about 70 μmol photons m^−2^ s^−1^ at the beginning of the first and 56 μmol photons m^−2^ s^−1^ at the beginning of the second cell cycle were both sufficient for division into four daughter cells (Table 1). Similarly, for division into eight cells, a sufficient mean light intensity was 332 μmol photons m^−2^ s^−1^ at the beginning of the first cell cycle and 274 μmol photons m^−2^ s^−1^ at the beginning of the second. This ability to divide into multiple daughter cells, even at low light intensities, allows the alga to grow and divide at high cell densities when the majority of incoming light is shaded [68,69], making it a highly efficient species for algal biotechnology [70,71]. Although the number of daughter cells produced was not affected, the different incident light intensities affected the timing of the reproduction processes, so that DNA replication and cell divisions in the second cell cycle were delayed by 4 and 2 h, respectively (Figure 1). Moreover, the daughter cells produced at the end of the second cell cycle were smaller than those at the end of the first cycle, and contained fewer proteins than the cells at the beginning of the experiment (Figure 2). As the RNA and starch contents per cell stayed approximately the same at 30 °C (Figure 2 and Figure 3), the cell composition was clearly altered by light intensity. Furthermore, cells at high light intensity were, in both cell cycles, richer in protein compared to the low-light-grown cells (Figure 2B). This phenomenon might be exploited when a protein-rich algal biomass is required [68,72]. Altogether, this documents the plasticity of the multiple fission lifestyle, which allows the cells to slightly prolong the growth phase in order to be able to reach a critical cell size for starting another reproductive sequence, and to maintain the reproduction potential at the expense of producing slightly smaller daughter cells with altered cell compositions. Such growth and metabolic plasticity of algae allows them to thrive both at low light intensities [68,69], and in the harsh conditions of a desert, with very high light intensity and extreme fluctuating temperatures [73,74,75,76]. 

Growth at the supra-optimal temperature led to a blockage of DNA replication and cell division (Figure 1), an approximately 30-fold increase in cell size (Figure 2D), and overaccumulation of both starch and lipids (Figure 3). The cell cycle arrest at supra-optimal temperatures has been established both in the related alga *Chlorella vulgaris* [18] and in the distantly related *Chlamydomonas reinhardtii* [17,77]. However, the nature of the blockage seems to be different in *P. kessleri*, since no DNA replication was detected (Figure 1), which is in contrast to both *Chlorella vulgaris* and *Chlamydomonas reinhardtii*, where (at least some) DNA replication occurred and the cell cycle blockage took place at the level of nuclear (and cellular) division(s) [17,18,77]. Indeed, it has been established that individual cell reproduction processes are sensitive to stress in an order opposite to their occurrence in the cell cycle, with cell division being the most sensitive process [16]. Thus, the observed behavior may also be caused by the supra-optimal temperature used, and if a lower temperature is used, DNA replication might not be affected. In the experiments presented, both supra-optimal temperatures of 39 °C and 40 °C caused the same blockage of cell division (Figure 1 and Figure 4), suggesting that the temperature of 40 °C is well above the threshold required to cause the cell cycle blockage. However, the effects of temperature are very precise and subtle, so a 1 °C variation in temperature can have the quite distinct effect of only a partial blockage [17,77]. Of the three effects of supra-optimal temperature treatment, the most striking was the approximately 32-fold overaccumulation of starch compared to the highest content reached in the control cells, and the approximately 508-fold increase compared to the initial cell content. The accumulation was very fast, as close to maximal values were reached within about 24 h at the high light intensity, and within about 36 h at the low light intensity (Figure 3A). At the optimal temperature, the net accumulation of starch was slower, and reached lower levels at the low light intensity; this phenomenon was even more pronounced at the supra-optimal temperature (Figure 3). This stresses the importance of light intensity for starch (over)production, and is consistent with the established knowledge in the field [38]. The starch overaccumulation at supra-optimal temperatures was consistent with the previous experiments on the related *Chlorella* sp. K [78], *Chlorella vulgaris* [18] and distantly related *Chlamydomonas reinhardtii* [17,77]. The overaccumulation of neutral lipids was delayed by about 16 hours compared with that of starch (Figure 3). This fits with the two-step production of starch, followed by neutral lipids, as stress conditions are prolonged [23,24,32,39]. Lipid production also proved to be light-dependent, as it was about six hours delayed at the low light intensity (Figure 3B). Although the lipids overaccumulated to about 64 times the initial values, their final cell content was much lower than that of starch (Figure 3), making starch the predominant component of the cell. Indeed, cell composition was strikingly affected by the supra-optimal temperatures. At the high light intensity, the cell volume (Figure 2D) increased about 30-fold, dry matter (Figure 2C) increased about 19-fold, RNA content more than 8-fold (Figure 2A), and protein content by about 32-fold (Figure 2B). Together with a massive 508-fold increase in starch, this made the biomass significantly enriched in starch and proteins. In general, starch (over)production can be attained either through increasing starch production by improving growth conditions (high light, high CO_2_ concentration) [36,37] or by decreasing starch consumption—mainly by blocking cell reproduction as the biggest starch consumer under nutrient-limiting conditions [24,25,26,32,39,41]. Indeed, a blockage of cell division has emerged as an effective tool to (over)produce starch [79]. Improved starch production by optimizing growth conditions, combined with decreasing its consumption due to inhibition of the major consumers, should lead to superior starch (over)accumulation. Rapid and extensive starch (over)production by supra-optimal temperature treatment was shown in the green alga *Chlamydomonas reinhardtii* in both laboratory-scale [17] and pilot-scale photobioreactors [38]. Compared to *Chlamydomonas reinhardtii, P. kessleri* grows at lower light intensities, and is able to reach very high cell densities [70,71]. Together with the ability to produce significant amounts of neutral lipids via a simple alteration of conditions, this makes it an excellent organism for algal biotechnology. The experiments presented here provide a means for the fast and reliable overproduction of starch by increasing the temperature to a supra-optimal one. Such a treatment is very simple, but a rapid and controllable increase in temperature, and maintenance of that temperature, might be complicated at a very large scale. Alternatively, the treatment can be used in closed pilot-scale reactors [38], where temperature control is more feasible. Such production would only be justifiable for high-added-value compounds, such as stable isotopically labeled ones.

The experiments on the combined effects of deuterium and temperature had a double rationale: Firstly, from a basic research point of view, it is intriguing to analyze the combination of deuterium, which increases bond dissociation and activation energies [46], with increased temperature, which is known to increase kinetic energy and, thus, speed up general metabolism. Secondly, (over)production of deuterated compounds such as starch, proteins, and/or neutral lipids is biotechnologically relevant, as these are very high-added-value products that can be exploited both as biochemical standards and in stable isotope-labeling experiments [43,52,53]. When starting the experiments, it was not clear which of the two phenomena would prevail. We assumed that the temperature increase might decrease the effect of the presence of deuterium, and at the same time that the deuterium might prevent the blockage of cell division observed in hydrogenated water at supra-optimal temperatures. As it turned out, some of our assumptions were confirmed, while others were not. Cultivation in increasing concentrations of deuterated water showed a concentration-dependent decrease in growth rates and cell division (Figure 4A,B). Interestingly, although the difference in growth rates between 30 °C and 35 °C in the control was negligible (Figure 4A, first panel), the cultivation at 35 °C promoted growth in all deuterated cultures (Figure 4A, second to fourth panels, green circles). This partially confirmed the prediction that increased temperature might alleviate the effects of deuterium on the cells. However, the effect was specific only to growth, and was not reflected in cell division (Figure 4B, green circles), as the cells remained large (Figure 4C, green circles). This implies that although the effects of deuterium on metabolism in general can be partially compensated for by temperature, the deuterium-induced problems with cell division are either more sensitive or more specific. A further increase in temperature to 39 °C inhibited both cell growth and division (Figure 4A,B, blue squares). The effect of supra-optimal temperatures was more detrimental to the deuterated cultures than to the controls, suggesting that the alleviating effect of 35 °C was replaced with a strictly stress effect. Due to the combination of the two stresses, the same temperature considered to be supra-optimal in control cultures might be above this threshold for highly deuterated cultures. Thus, temperature in deuterated cultures showed aspects of hormesis—a phenomenon where low concentrations of a compound are beneficial but higher levels cause stress [80,81]. The significant stress caused to the deuterated cells by the supra-optimal temperatures was further supported by a rapid decrease in the Fv:Fm ratio in all deuterated cultures after about 36 h of the experiment (Figure 4D, second to fourth panels, blue squares). This was in contrast to the control cultures at the same temperatures (Figure 4D, blue squares), which were not significantly affected. The cultures of *P. kessleri* showed striking resilience to the presence of deuterium, as they were not significantly stressed by growth in very high deuterated water concentrations for more than three days (Figure 4D, yellow and green lines). Only on the fourth day of cultivation did the cultures at the two highest deuterated water concentrations (90% and 99%) start to be stressed (Figure 4D, third and fourth panels). However, the absence of cell division at the higher deuterated water concentrations allowed (over)accumulation of starch, although the temperature did not seem to have a promoting or inhibiting effect in such cultures (Figure 5, compare B–D with J–L). The combination of supra-optimal temperatures and growth in deuterated water promoted the accumulation of neutral lipids (Figure 5, panels N–P). These experiments confirmed that the effect of supra-optimal temperatures is a general phenomenon not only in different organisms [17,18,77], but also in different growth conditions. If desired, such treatment can be used to (over)produce deuterated starch and, interestingly, also deuterated neutral lipids. Due to limitations in maintaining supra-optimal temperatures and the inhibitively high prices of deuterated water, such treatment is conceivable only at small scales for the production of stable isotopically labeled compounds [43,52,53]. This is consistent with the proposed algal production of deuterated sugars and amino acids [58], proteins [59], chlorophylls [52,60], and carotenoids [52]. Supra-optimal temperature seems to be a promising tool for a rapid increase in starch (and neutral lipid) content, compared to the slower effect of nutrient starvation [43]. Furthermore, it offers recovery of other deuterated cell components.

## 5. Conclusions

We describe the effects of supra-optimal temperatures on synchronized cultures of the green alga *P. kessleri.* Supra-optimal temperatures block DNA replication and cell division, but do not affect cell growth. Moreover, due to the absence of cell division, starch and neutral lipids overaccumulate to levels 508-fold and 64-fold those of the initial values in the daughter cells. This leads to the production of large cells with altered cell compositions, consisting mostly of proteins and starch. We tested whether increased cultivation temperatures could alleviate the effects of deuterium in heavy water. Temperature showed a hormetic effect, as a temperature increase over optimum improved cell growth, but the supra-optimal temperatures caused extensive stress. The cultures were strikingly resilient to the stress caused by heavy water, and were able to grow and divide even at 90% D_2_O, although they were only able to grow but not divide at 99% D_2_O. The supra-optimal temperature treatment, when applied to cultures grown in heavy water, had similar effects as in normal water, and led to the production of cells with increased starch and neutral lipid contents. Our results establish new treatments for the overaccumulation of starch (and proteins) in *P. kessleri*, and they also prove that such treatment can be used to produce deuterated starch, proteins, and neutral lipids for biotechnological purposes. 

## Figures and Tables

**Table 1:** Changes in mean light intensities experienced by the cultures during the experiment at different incident light intensities and temperatures.

Mean Light Intensity (μmol photons m^−2^ s^−1^)					
Incident Light Intensity		110 μmol photons m^−2^ s^−1^		500 μmol photons m^−2^ s^−1^	
Temperature		30 °C	40 °C	30 °C	40 °C
**Time (h)**	0	70 ± 5	75 ± 5	332 ± 15	332 ± 12
	24	56 ± 5	54 ± 7	274 ± 10	261 ± 10
	42	50 ± 5	50 ± 5	186 ± 10	220 ± 10
	48	50 ± 5	50 ± 5	177 ± 10	220 ± 10

**Table 2:** Comparison of mass and cell number doubling times for cultures grown in media containing different concentrations of deuterated water at different temperatures. The average of at least three experiments is shown.

Concentration of Deuterated Water (%)	Temperature (°C)	Mass Doubling Time (h)	% of Control	Cell Number Doubling Time (h)	% of Control
0	30	9.58	100.00	8.85	100.00
	35	10.66	100.00	9.99	100.00
	39	28.18	100.00	24.84	100.00
70	30	22.97	239.67	14.18	160.25
	35	17.01	159.54	28.90	289.35
	39	36.48	129.46	63.35	255.00
90	30	32.23	336.30	21.00	237.37
	35	24.01	225.27	36.35	363.91
	39	43.92	155.86	73.63	296.38
99	30	42.34	441.87	35.35	399.54
	35	35.32	331.37	92.33	924.32
	39	50.61	179.58	63.51	255.66

## Data Availability

All data presented in this study are available within this article. There are no special databases associated with this manuscript.
